# Hæmatoma—Recurrent Tumor in the Ox

**Published:** 1890-01

**Authors:** 


### HÆMATOMA—RECURRENT TUMOR IN THE OX.

BY DR. NARIMAN.

The Quarterly Journal of Veterinary Science, India, Oct., 1889.

A red bullock in good condition was admitted in the Bai Sakerbai Dinshaw Petit Hospital on 20th of May, 1889, for a round tumor double in size to a cocoanut on the left side of the chest extending from the 5th to 7th ribs, said to have been caused by a bale of cotton falling on the part. The diameter of the tumor was a little more than five inches. The tumor was hard, slightly fluctuating in the centre, and firmly adherent at the bottom, but not at all tender on pressure. The case being considered one of hæmatoma, an incision was made at the lower part of the tumor, but only a very little serous fluid escaped. A seton was then passed through the same opening and brought out at the upper part of the tumor; this was retained for a week being dressed every day with turpentine ointment. This did not produce the desired effect, as only slight portions of the tumor near the openings sloughed, while the whole mass of the tumor was as hard as before. On June 22d, the animal was cast, an elliptical incision about seven inches long and four inches at its broadest part was made from above downwards, and the growth dissected out. The tumor consisted of large masses of tough lymph, portions of which appeared quite striated and fibrous. The bleeding was profuse, which prevented thorough removal; after checking the bleeding as much as possible, the wound was dressed with pure carbolic acid. The tumor began to grow with vigor, so that in about two weeks' time it was as large as before; this time it looked like masses of muscle though it was quite pale. The growth was then removed a second time taking sufficient care to dissect out all nodular masses adherent to the muscle underneath, and the same dressing was applied. The case then progressed favorably except that there were small growths sprouting at three places; these were nipped out completely and the wound dressed with carbolic acid. The case then made rapid recovery, the whole gap filled up except a portion at the supero-anterior part where the sinus formed, which also healed under injections of strong carbolic solution. The case was discharged cured on the 21st of July, with a very small longitudinal scar, notwithstanding the fact that the incision removed about four inches of skin.

It is interesting to note the following points about the case :—

1. That lymph or blood exuded in a bruised part is liable to remain for a long time and become converted into a lowly organized fibrous tumor.

2. That the tumor was so large and yet free from all deeper structures, except being connected here and there with muscles, so that during dissection neither ribs nor pleura were exposed.

3. That the tumor, although composed entirely of lymph, was very vascular, the blood lost at the first operation being more than a quart.

4. That the tumor, though benign, recurred as long as all masses of lymph were not completely removed, thus appearing to a casual observer as a malignant growth.

